# Mental Fatigue and Functional Near-Infrared Spectroscopy (fNIRS) – Based Assessment of Cognitive Performance After Mild Traumatic Brain Injury

**DOI:** 10.3389/fnhum.2019.00145

**Published:** 2019-05-14

**Authors:** Simon Skau, Lina Bunketorp-Käll, Hans Georg Kuhn, Birgitta Johansson

**Affiliations:** ^1^Institute of Neuroscience and Physiology, Sahlgrenska Academy, University of Gothenburg, Gothenburg, Sweden; ^2^Centre for Advanced Reconstruction of Extremities, Sahlgrenska University Hospital, Mölndal, Sweden; ^3^Department of Neurology, Center for Stroke Research, Charité – Universitätsmedizin, Berlin, Germany

**Keywords:** mild TBI, fNIRS, mental fatigue, frontal cortex, conflict processing, processing speed

## Abstract

Pathological mental fatigue after mild traumatic brain injury (TBI-MF) is characterized by pronounced mental fatigue after cognitive activity. The neurological origin is unknown, and we aimed in the present study to investigate how prolonged mental activity affects cognitive performance and its neural correlates in individuals with TBI-MF. We recruited individuals with TBI-MF (*n* = 20) at least 5 months after injury, and age-matched healthy controls (*n* = 20). We used functional near-infrared spectroscopy (fNIRS) to assess hemodynamic changes in the frontal cortex. The self-assessed mental energy level was measured with a visual analog scale (VAS) before and after the experimental procedure. A battery of six neuropsychological tests including Stroop–Simon, Symbol Search, Digit Span, Parallel Serial Mental Operation (PaSMO), Sustained Attention and Working Memory test, and Digit Symbol Coding (DSC) were used. The sequence was repeated once after an 8 min sustained-attention test. The test procedure lasted 2½ h. The experimental procedure resulted in a decrease in mental energy in the TBI-MF group, compared to controls (interaction, *p* < 0.001, η_p_^2^ = 0.331). The TBI-MF group performed at a similar level on both DSC tests, whereas the controls improved their performance in the second session (interaction, *p* < 0.01, η_p_^2^ = 0.268). During the Stroop–Simon test, the fNIRS event-related response showed no time effect. However, the TBI-MF group exhibited lower oxygenated hemoglobin (oxy-Hb) concentrations in the frontal polar area (FPA), ventrolateral motor cortex, and dorsolateral prefrontal cortex (DLPFC) from the beginning of the test session. A Stroop and Group interaction was found in the left ventrolateral prefrontal cortex showing that the TBI-MF group did have the same oxy-Hb concentration for both congruent and incongruent trials, whereas the controls had more oxy-Hb in the incongruent trial compared to the congruent trial (interaction, *p* < 0.01, η_p_^2^ = 0.227). In sum these results indicate that individuals with TBI-MF have a reduced ability to recruit the frontal cortex, which is correlated with self-reported mental fatigue. This may result both in deterioration of cognitive function and the experience of a mental fatigue after extended mental activity.

## Introduction

Traumatic brain injury (TBI) is a common neurological condition affecting people of all ages. Globally, approximately 295/100,000 people are treated in hospitals each year for TBI (Nguyen et al., 2016). This is mild for most patients who recover within 1–3 months (Carroll et al., 2004). For those who show insufficient recovery within this period, fatigue is common (King et al., 1995; Kraus et al., 2014; McInnes et al., 2017), irrespective of injury severity (Johansson et al., 2009). Long-lasting mental fatigue interferes considerably with daily living, the ability to work and has a negative impact on well-being and mental health (Cantor et al., 2008; Hawthorne et al., 2009; Johansson et al., 2009; Lannsjo et al., 2009; Ahman et al., 2013). Fatigue is also suggested to be a direct consequence of TBI and not a result of depression, pain or sleep disturbances (Cantor et al., 2008). Due to the current limitations in measuring fatigue and the complexities involved in objectively measuring fatigue, researchers frequently rely on subjective questionnaire-based reporting scales, both for the physical and the mental components.

Pathological mental fatigue (MF) is characterized by mental exhaustion during sensory stimulation or extended cognitive activity, and with a disproportionately long recovery. MF is a typical symptom in many neurological diseases (Lindqvist and Malmgren, 1993; Johansson et al., 2010). The underlying origin of MF is unknown, but it is suggested to be related to circuits that connect basal ganglia, amygdala, the thalamus and frontal cortex (Chaudhuri and Behan, 2004). These circuits mediate motivation, learning, planning, goal-directed behavior and emotion regulation. The integration of networks is important for appropriate behavior and cognitive functioning (Haber and Calzavara, 2009). Studies of MF after moderate and severe TBI using functional magnetic resonance imaging (fMRI) indicate a dysfunction within the cortico-striatal-thalamic circuits (Kohl et al., 2009; Nordin et al., 2016; Berginstrom et al., 2017; Moller et al., 2017; Wylie et al., 2017).

Cognitive impairment associated with MF after TBI has been related to reduced processing speed and attention (Park et al., 1999; Azouvi et al., 2004; Ziino and Ponsford, 2006a,b; Ashman et al., 2008; Belmont et al., 2009; Johansson et al., 2009, 2010; Ponsford et al., 2011), these being the cognitive functions most susceptible to brain injury (Frencham et al., 2005; Lannsjo et al., 2009). In our previous study, investigating individuals suffering from MF after acquired brain injuries, impaired cognitive performance in processing speed (Digit Symbol Coding) (Wechsler, 2010), attention (SAWM) (Johansson and Ronnback, 2015), attentional blink (Dux and Marois, 2009), and working memory (Digit Span) (Wechsler, 2010) over a 2 h test period was found, with no improvement for those suffering from MF whereas the controls improved (Jonasson et al., 2018). Similar results have been reported in additional studies (Ashman et al., 2008). These studies indicate that people suffering from MF after an acquired brain injury find it challenging to repeat cognitive tasks, whereas improvement can be achieved for healthy controls.

In this study, we aimed to explore how prolonged mental activity affects cognitive performance as well as exploring the neural correlates of conflict processing in individuals with MF after TBI (TBI-MF). This was done with a block of six neuropsychological tests including Stroop–Simon (Egner et al., 2007), Symbol Search (Wechsler, 2010), Digit Span (Wechsler, 2010), Parallel Serial Mental Operation (PaSMO) (Reitan and Wolfson, 1985), SAWM (Johansson and Ronnback, 2015), and Digit Symbol Coding (DSC) (Wechsler, 2010), were performed. The sequence was repeated once. Between the two blocks, an 8 min sustained-attention test and the self-report of MFS was done, with the intention to induce even more fatigue. The test procedure lasted for approximately 2½ h.

To measure brain activity, functional near-infrared spectroscopy (fNIRS) was used. Similar to fMRI, fNIRS measures the hemodynamic response to neural activity. fNIRS is a noninvasive optical brain imaging technique that uses near-infrared light to measure the relative concentration of oxygenated (oxy-Hb) and deoxygenated (deoxy-Hb) hemoglobin (Jo Bsis-Vandervliet, 1999). By applying light emitting sources on the scalp the fNIRS can detect hemodynamic change that occur to a depth of 1.5–2 cm into the cortex (Jo Bsis-Vandervliet, 1999; Obrig, 2014). Compared to other imaging techniques, fNIRS is more robust to movement artifacts (Balardin et al., 2017). Thus, fNIRS is well-suited to studies in natural environments (Kopton and Kenning, 2014), and has been used in psychiatric studies (Ehlis et al., 2014), and for cognitive assessment (Irani et al., 2007; Ferrari and Quaresima, 2012; Ehlis et al., 2014). MF has been investigated by means of fNIRS in patients with multiple sclerosis, and the level of self-reported fatigue correlated with neural activity in the dorsolateral prefrontal cortex (DLPFC) during a working memory task (Borragán et al., 2018). To the best of our knowledge, no previous study has used fNIRS to study MF resulting from TBI. There are, however, a few cognitive studies using fNIRS in subjects with TBI. Most of these studies are exploratory, with few participants (Merzagora et al., 2011; Hibino et al., 2013; Kontos et al., 2014; Rodriguez Merzagora et al., 2014; Sawamura et al., 2014; Helmich et al., 2015; Plenger et al., 2016). With the exception of one study (Rodriguez Merzagora et al., 2014), fNIRS measurements show decreased activity in the DLPFC for the TBI group compared to controls for cognitive performance (Merzagora et al., 2011; Hibino et al., 2013; Kontos et al., 2014; Sawamura et al., 2014; Helmich et al., 2015; Plenger et al., 2016). Other studies on healthy adults applying fNIRS while performing the conflict processing Stroop test have reported increased activity in the DLPFC (Schroeter et al., 2002, 2004; Leon-Carrion et al., 2008; Hyodo et al., 2012; Endo et al., 2013; Lague-Beauvais et al., 2013; Byun et al., 2014; Yasumura et al., 2014). We opted to investigate brain activity using the Stroop–Simon task (Egner et al., 2007) adapted to fNIRS in a cohort with TBI-MF.

We hypothesized that, during performance of the Stroop–Simon test, the TBI-MF group would show less activity in the DLPFC, as compared to controls. In addition, individuals with TBI-MF may demonstrate altered brain activity in the frontal cortex between the first and second test sessions. This hypothesis was not directed, meaning that we did not know based on previous literature if the alteration would be an increased or decreased in oxygen consumption, as compared to controls. We also hypothesized that the TBI-MF group would perform less well in the second test session, as compared to the first.

## Materials and Methods

### Study Participants and Protocol

Twenty individuals with long-term TBI-MF (minimum 5 months after injury) were recruited from the Department of Neurology, Sahlgrenska University Hospital, Gothenburg. Inclusion criteria were as follows: diagnosed with mild TBI according to the definition proposed by The World Health Organization Collaborating Centre for Neurotrauma Task Force (Carroll et al., 2004); scoring above the cut-off score of 10.5 on the Mental Fatigue Scale (MFS) (Johansson and Rönnbäck, 2014); aged 20–65 years and not suffering from any other psychiatric or neurological disorders. All participants had recovered well, were independent in their daily living, with the exception of their prolonged MF. Twenty-one healthy controls who neither suffered from MF (below 10.5 points on MFS), nor did they have any psychiatric or neurological disorders, were recruited at the request of the general community. The study was approved by the regional Ethical Review Board in Gothenburg (reference number: 028-16). The participants gave their informed written consent before the assessment and were told that they could withdraw at any time.

### Experimental Design

**FIGURE 1:**
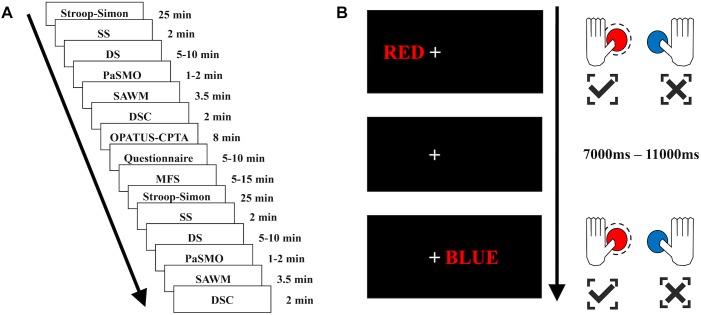
Test sequence and illustration of the Stroop–Simon test. **(A)** The test sequence consisted of 6 tests – Stroop–Simon, SS (Symbol Search), DS (Digit Span), PaSMO (Parallel Serial Mental Operation), SAWM (Speed, divided Attention and Working Memory), DSC (Digit Symbol Coding) – which were repeated once after the OPATUS-CPTA (one-back sustained attention task), and MFS, Mental Fatigue Scale questionnaire were applied. The time indicates the length of each test, not including practice and preparation for each test. **(B)** Examples of stimulus-responses for the Stroop–Simon test. When a word written in red color is presented, the correct response is to press the left button (marked in red on the game pad), irrespective of the placement (Simon effect – left or right) or meaning of the word (Stroop effect – “RED” or “BLUE”). Both examples (top and bottom) require to press the left (red) button. Top example: a Stroop(word)-congruent/Simon(place)-congruent stimulus, bottom example: a Stroop(word)-incongruent/Simon(place)-incongruent stimulus. All stimuli were presented 7,000–11,000 ms apart.

The participant was seated in a chair next to a table with a computer screen. All tests were performed sitting in the same location, whereas the tasks differed with respect to the response type, such as pen and paper task, verbal response tasks or computerized tasks where the participant used the computer mouse, tablet or a gamepad. The fNIRS cap with optodes attached was carefully placed on the participant’s head and was worn throughout the whole experimental sessions. To minimize ambient light reaching the scalp during the test procedure, the fNIRS cap was covered by another stretchable cap. The experiment consisted of two identical test sessions with 6 individual tests performed in the same sequence (Figure 1A): (1) Stroop–Simon (Egner et al., 2007); (2) Symbol Search (SS, WAIS-IV) (Wechsler, 2010); (3). Digit Span (DS, WAIS-IV) (Wechsler, 2010); (4) Parallel Serial Mental Operations (PaSMO) (Reitan and Wolfson, 1985); (5) a computerized test combining Speed, divided Attention and Working Memory simultaneously (SAWM) (Johansson and Ronnback, 2015), and (6) Digit Symbol Coding (DSC, WAIS-IV) (Wechsler, 2010). The two sessions were separated by once presenting a sustained-attention test with an 8 min one-back task (OPATUS-CPTA) and completing the MFS (Figure 1A). MFS is invariant to age, gender and education (Johansson et al., 2010; Johansson and Ronnback, 2014). In total, the test procedure took 2½ h. Participants were allowed to take a short break and drink water or stand up and stretch their legs between tests, while keeping the fNIRS cap on. Before start of the experimental procedure and after completion of all tests, the participants were asked to rate their energy level on a visual analog scale (VAS). They specified their energy level on a continuous line (10 cm) indicating a position between the two end-points, “full of energy” and “totally exhausted, no energy left.”

### Cognitive Tests

The Stroop–Simon test was used to measure both the Stroop effect (conflict between semantic meaning and ink color) and the Simon effect (conflict between stimulus location and response hand) (Egner, 2008; Forster and Cho, 2014). The participant was asked to fixate on the cross located in the middle of the screen. The word RED or BLUE – either in red or blue ink – was presented to the left or right side of the cross (5 cm). The participant was asked to respond to the ink color by pressing a button on a gamepad with either the left thumb for red, or the right thumb for blue (see Figure 1B). The response to stimulus interval was randomly assigned to be between 7 and 11 s, and the participants had 3 s to respond. This created four different types of trials and each trial had two dimensions; Stroop congruent and Simon congruent (CC), Stroop congruent and Simon incongruent (CI), Stroop incongruent and Simon congruent (IC), or Stroop incongruent and Simon incongruent (II) (see Figure 1B). There was a total of 164 stimuli, semi-randomized with a 30 s pause after half of the stimuli. Reaction time, errors made and omissions were used as raw scores.

Symbol Search (SS) and Digit Symbol Coding (DSC) are subtests within the Processing Speed Index in WAIS-IV (Wechsler, 2010) that were used to measure attention, speed of mental and psychomotor operation and visual discrimination. In both tests, the subject is asked to perform as many symbols as possible during 2 min. Raw score is the number of correct symbols performed.

Digit Span (DS) from WAIS-IV (Wechsler, 2010) was used to assess working memory. Raw scores were reported as the number of correctly repeated strings of digits.

The PaSMO (Reitan and Wolfson, 1985) was used to measure mental control and tracking in a task similar to the Trail Making Test (Reitan and Wolfson, 1985). In PaSMO, the participants were asked to say the whole alphabet with the corresponding digits, i.e., A1, B2, C3, and so forth, as fast as possible. Performance was measured in time (seconds) with a faster time indicating a better performance.

The SAWM test was used for simultaneous assessment of Speed, divided Attention and Working Memory (Johansson and Ronnback, 2015). The test measured number of mouse clicks in four squares, located in each corner of a larger square on the computer screen, and was performed in a clockwise order. At the same time, the subject was asked to count how many instances of a specific digit were shown (seen in the square located to the upper right). Another digit, between zero and nine was randomly chosen for each run. The digits to be counted were randomly displayed for 1 s in a square located to the upper left. After 30 s, the subject was asked to report how many of the specific digits he/she had seen. The answer was compared with the correct number of the digits presented, and the numbers of errors made in each run was analyzed. The number of clicks was simultaneously recorded. Each session lasted for 30 s and was repeated five times. All participants had the opportunity to rehearse the task before starting the assessment to ensure that they had understood the task instructions displayed on the computer screen. The raw scores used for analysis were the mean scores of errors and number of successful responses from the second to fifth run.

The continuous performance test with a one-back task (OPATUS-CPTA) was used as a measure of sustained attention. The 8 min OPATUS-CPTA task was delivered on a mini tablet, and the participant was asked to tap the screen when the same symbol as the one previously shown appeared on the screen. Triangles pointing up/down/left/right and appeared in two colors: yellow or blue, stimuli duration was 150 ms, inter stimuli interval was 2000 ms, target rate was 20% and the number of trials was 240. Reaction time, errors made and omissions were used as raw scores.

### fNIRS Data Acquisition

**FIGURE 2:**
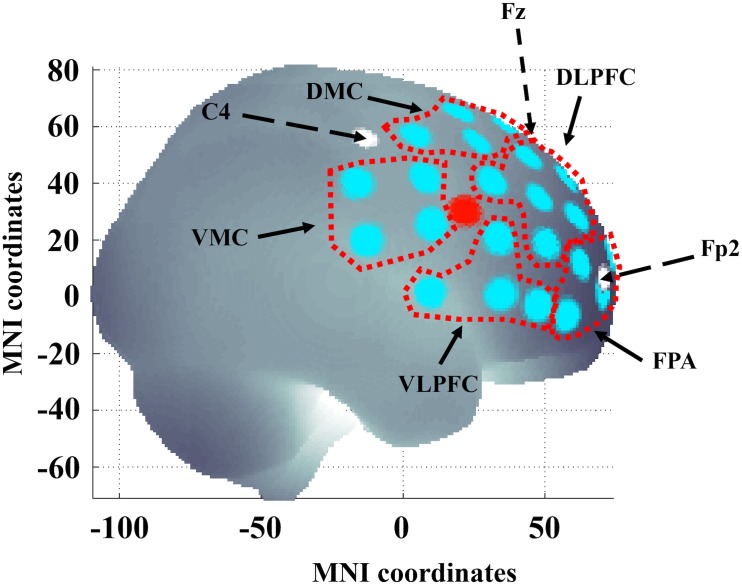
Channel locations in the right hemisphere. Areas delineated with a red dotted line indicate the regions of interest (ROIs): left and right frontal polar area (FPA) corresponding to Brodmann area (BA) 10; dorsolateral prefrontal cortex (DLPFC) corresponding to BA 9 and 46; dorsal motor cortex (DMC) corresponding to BA 6 and 8; ventrolateral prefrontal cortex (VLPFC) corresponding to BA 44 and 45 and ventral motor cortex (VMC) corresponding to BA 6 and 44. The large red dot indicates the location of the short separation channel. Dashed black arrows and white dots indicate 10/20 reference points C4, Fp2, and Fz. The brain template was generated from a MATLAB-based toolbox (Singh et al., 2005). x- and y-axis refer to MNI (Montreal Neurological Institute) coordinate system.

The fNIRS measurements were performed using a continuous wave system (NTS) Optical Imaging System, Gowerlabs Ltd., United Kingdom (Everdell et al., 2005) using two wavelengths (780 and 850nm) to measure changes in the concentration of oxygenated hemoglobin (oxy-Hb), deoxygenated hemoglobin (deoxy-Hb) and their sum total hemoglobin (tot-Hb). The system has 16 dual-wavelength sources and 16 detectors. The array used provided 50 standard fNIRS channels (i.e., source/detector pairs) plus two short-separation channels. There were 44 channels with a source-detector distance of 30 mm and 6 channels with 45 mm distance. The six longer channels connected the two hemispheres, but the signal quality was too low for the data to be included in our analysis. The distance for the short-separation channels was 10 mm, as suggested in previous studies (Gagnon et al., 2011; Brigadoi and Cooper, 2015). Short separation channels are only sensitive to hemodynamics in the scalp. Since the regular separation channels measure signals originating in both the brain and the scalp, the use of short-separation channels allowed us to regress out the scalp signal from regular-separation signals with the aim to improve the brain specificity of the fNIRS measurement (Gagnon et al., 2011; Brigadoi and Cooper, 2015). The placement of the optodes was designed to encompass five regions of interest (ROIs) on each hemisphere covering areas of the frontal cortex, previously reported to be involved in executive function and cognitive control tasks (Roberts and Hall, 2008). The ROIs were left and right frontal polar area (FPA) or Brodmann area (BA) 10; DLPFC or BA 9 and 46; dorsal motor cortex (DMC) BA 6 and 8; ventral lateral prefrontal cortex (VLPFC) BA 44 and 45 and ventral motor cortex VMC BA 6 and 44 (see Figure 2). Data were acquired at a sampling frequency of 10 Hz.

### fNIRS Data Analysis

The fNIRS data were preprocessed using MATLAB 2014a (MATLAB, 2014) and the MATLAB based fNIRS-processing package HomER2 (Huppert et al., 2009). The processing pipeline started with pruning the raw data such as that channels were rejected if their mean intensity was below the noise floor of the instrument (5e-4 A.U.). The raw data was then converted to optical density (OD). The HomER2 functions enPCAFilter, hmrMotionArtifact and hmrMotionCorrectSpline were used to correct for motion artifacts. A high band-pass filter of 0.03 was used to correct for drift and a low band-pass 0.5 filter to remove pulse and respiration. To calculate the hemodynamic response function (HRF) the GLM_HRF_Drift_SS function in HomER2, which estimates the HRF by applying a General Linear Model (GLM), was used. To solve the GLM, a least square fit of a convolution model in which the HRF at each channel and chromophore was modeled as a series of Gaussian basis function, with a spacing and standard deviation of 0.5 s (Ye et al., 2009). The model included polynomial drift regressors up to the 3rd order. The regression time length was −2–12 s. The short channels selected for regression for each long channel was those with the highest correlation to the regular channel. The analysis of the Stroop–Simon data was analyzed with pre-determined ROIs. A visual analysis of each channel was done and the channels that remained too noisy were removed, but not to the exclusion of any ROI. On average, 0.65 channels per ROI was excluded after the pruning function and manual removal.

Since the combination of wavelengths used (780, 850 nm) is more sensitive to oxy-Hb compared to deoxy-HB, only the oxy-Hb data were statistically analyzed (Uludag et al., 2004; Sato et al., 2013). The deoxy-HB did not add any further information to this study and is not included. For the Stroop–Simon test the maximum peak between 3 and 9 s after each stimulus was identified. One second around the peak value was averaged.

### Statistics

A two-way repeated ANOVA with within-subject factor *Time* (first and second test session) and the between-subject factor *Group* for each particular test was used. For items having both a group and an interaction effect, independent *t*-tests as *post hoc* test were used for interpreting the main result. For the Stroop–Simon test a four-way repeated ANOVA was performed with the following within-subject factors; (i) Time (first and seconds sessions); (ii) Stroop (congruent and incongruent); (iii) Simon (congruent and incongruent); and (iv) the between-subject factor Group (patients and controls). For the Stroop–Simon test, mean reaction time was used for each stimulus type for each participant. This excluded omissions (more than 3 s after stimulus without an answer), error trials, the trial set after the error and condition-specific outlier values that were greater than 2 SDs from the mean.

fNIRS data acquired during the Stroop–Simon test were analyzed within each ROI and only for the Stroop effect. A three-way repeated ANOVA with within-subject factor, Time (first and second test sessions) and Stroop (congruent and incongruent) and the between-subject factor, Group for each ROI was used. The CC trials were used as congruent and the average of the IC and II trials were used as incongruent. *T*-test, chi-square and Pearson’s correlation was used for demographic data, *post hoc* analysis and OPATIS-CPTA. To correct for multiple comparisons with the neuropsychological test and the fNIRS, a false discovery rate (FDR) was used with the *q*-value set to 0.05 in order to keep the false positive rate at 5% (Singh and Dan, 2006). Parametric tests were done using SPSS version 25 and Matlab statistical toolbox (MATLAB, 2014). The datasets generated in the current study are available from the corresponding author on reasonable request.

## Results

### Demographics

Demographical and clinical characteristics of the study population are presented in Table 1. One control subject was excluded due to failure to follow instruction to remain seated and physically calm during the fNIRS session. The mean time since injury was 28 (±21) months. The only variable that differed significantly between groups was the MFS score, that was rated significantly higher in the TBI-MF group as compared to controls (*p* < 0.001). No correlation between time since injury and MFS was found (*r* = 0.21, *p* = 0.35). Eight of the individuals with MF-TBI (40%) had experienced two or more mild TBI, but no significant difference was found in any tests or rating on MFS in these individuals compared to those who had suffered only one mild TBI. Six individuals with TBI-MF (30%) received methylphenidate drug treatment but had suspended the treatment 1 week prior to the assessment. No significant differences with respect to the cognitive test results and ratings on MFS were detected between these six individuals compared to the other individuals with TBI-MF.

**Table 1:** Background data for the TBI-MF and the control group.

	TBI-MF group	Control group	*p*-values
Age (years)	42.1 ± 10.2	39.3 ± 11.9	0.285
Range (years)	24–64	24–61	
Sex, females/males (n)	13/7	12/8	0.744
MFS score	21.3 ± 5.4	3.3 ± 2.9	<0.001
Education, upper secondary school/university (n)	3/17	4/16	0.677
Time elapsed since TBI exposure (months)	27.8 ± 21.2		
Range (months)	5–85		
Exposed to one/more than one mild TBI (n)	12/8		
**Employment status at time of the study (n)**			
100%	1	19	
75%	1	0	
50%	7	1 (of free choice)	
25%	1	0	
0%	10	0	
**Preinjury employment status (n)**			
100%	19		
50%	1		
Have undergone neuropsychological testing previously (Yes/No)	8/12	6/14	0.520

*Data are presented as Mean ± SD unless otherwise noted.*

**FIGURE 3:**
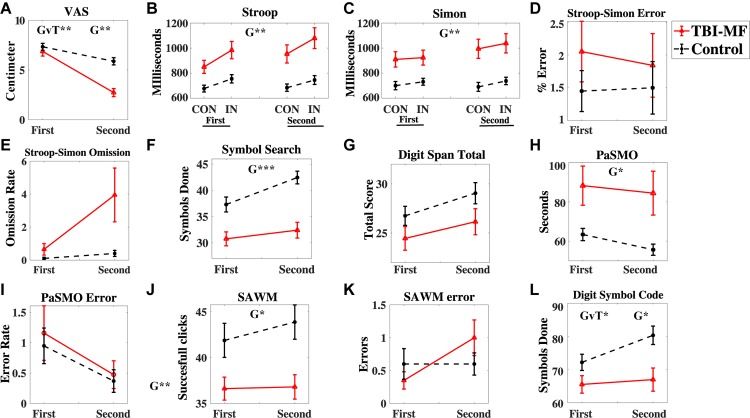
Behavioral test results. **(A)** Energy level measured by the VAS scale (0–10 cm visual analog scale), **(B–E)** Stroop-Simon test, **(B)** Stroop effect reaction time, **(C)** Simon effect reaction time, **(D)** Error rate Stroop-Simon, **(E)** Omission rate Stroop–Simon, **(F)** Symbol Search – number of correct symbols done, **(G)** Digit Span total score, **(H)** PaSMO performance in seconds, **(I)** PaSMO – number of errors, **(J)** SAWM total successful responses, **(K)** SAWM – number of errors, **(L)** Digit Symbol Coding – number of correct symbols done. In **(B)** *CON* represent the congruent stimuli, and *IN* the incongruent stimuli. Both stimulus types are illustrated in Figure 1B and described in the Materials and Methods section. Error bars represent standard error of mean. Significant effects are indicated for Group (G) and Group-Time interaction (GvT). ^∗∗∗^FDR adjusted *p* < 0.001, ^∗∗^FDR adjusted *p* < 0.01, ^∗^FDR adjusted *p* < 0.05.

A significant interaction effect was detected with respect to the subjective experience of mental energy (Table 2). Prior to start of the experiment the experienced energy level did not differ between groups (*t* = 0.792, FDR adjusted *p* > 0.05, with a Cohen’s *d* of 0.26), whereas after the experiment, the TBI-MF group rated their energy level significantly lower than controls (*t* = 5.769, FDR adjusted *p* < 0.05, with a Cohen’s *d* of 1.37) (Figure 3A).

**Table 2:** Repeated ANOVA for the behavioral test results.

	TBI	Control	Df	Time vs. Group FDR |*F* | η_p_^2^	Group FDR |*F* | η_p_^2^
VAS (cm)			1,36	FDR = ^∗∗^	FDR = ^∗∗^
Before	3.13 (2.0)	2.66 (1.5)		*F* = 17.812	*F* = 14.912
After	7.27 (1.7)	4.12 (1.6)		η_p_^2^ = 0.331	η_p_^2^ = 0.293
Stroop–Simon RT			1,37	FDR = ns	FDR = ^∗∗^
Test 1	918 (271)	717 (143)		*F* = 7.685	*F* = 12.117
Test 2	1019 (345)	716 (157)		η_p_^2^ = 0. 172	η_p_^2^ = 0.247
Stroop–Simon error			1,37	FDR = ns	FDR = ns
Test 1	0.02 (0.02)	0.01 (0.01)		*F* = 0.139	*F* = 0.714
Test 2	0.02 (0.02)	0.02 (0.02)		η_p_^2^ = 0.004	η_p_^2^ = 0.019
Stroop–Simon			1,37	FDR = ns	FDR = ns
omission Test 1	0.65 (1.5)	0.1 (0.3)		*F* = 4.867	*F* = 4.933
Test 2	3.95 (7.3)	0.4 (0.8)		η_p_^2^ = 0.116	η_p_^2^ = 0.118
Symbol search (SS)			1,38	FDR = ns	FDR = ^∗∗∗^
Test 1	30.8 (5.9)	37.3 (6.4)		*F* = 5.697	*F* = 21.343
Test 2	32.4 (6.7)	42.5 (5.4)		η_p_^2^ = 0.130	η_p_^2^ = 0.360
Digit span (DS)			1,38	FDR = ns	FDR = ns
Test 1	24.4 (5.4)	26.8 (4.3)		*F* = 0.589	*F* = 2.707
Test 2	26.2 (5.9)	29.0 (4.8)		η_p_^2^ = 0.015	η_p_^2^ = 0.066
PaSMO			1,36	FDR = ns	FDR = ^∗^
Test 1	88.2 (43.3)	63.4 (13–6)		*F* = 0.402	*F* = 6.471
Test 2	84.4 (48.6)	55.7 (12.4)		η_p_^2^ = 0.011	η_p_^2^ = 0.152
PaSMO error			1,36	FDR = ns	FDR = ns
Test 1	1.16 (2.0)	0.95 (1.3)		*F* = 0.041	*F* = 0.193
Test 2	0.47 (1.0)	0.37 (0.8)		η_p_^2^ = 0.001	η_p_^2^ = 0.005
SAWM			1,38	FDR = ns	FDR = ^∗^
Test 1	36.6 (5.6)	41.9 (8.3)		*F* = 2.099	*F* = 7.996
Test 2	36.8 (6.0)	43.9 (8.4)		η_p_^2^ = 0.052	η_p_^2^ = 0.174
SAWM error			1,38	FDR = ns	FDR = ns
Test 1	0.35 (0.6)	0.60 (1.0)		*F* = 3.055	*F* = 0.107
Test 2	1.00 (1.0)	0.60 (0.8)		η_p_^2^ = 0.074	η_p_^2^ = 0.003
DSC			1,38	FDR = ^∗^	FDR = ^∗^
Test 1	65.6 (11.7)	72.2 (10.9)		*F* = 13.942	*F* = 6.423
Test 2	67.0 (15.6)	80.4 (12.4)		η_p_^2^ = 0.268	η_p_^2^ = 0.145

*VAS, Energy level on a visual analog scale of 0–10 cm; RT, reaction time; PaSMO, Parallel Serial Mental Operation; SAWM, Speed, divided Attention and Working Memory; DSC, Digit Symbol Coding, FDR, p-value after adjustment with false discovery rate; ^∗∗∗^p < 0.001, ^∗∗^p < 0.01, ^∗^p < 0.05, ^ns^p > 0.05; η_p_^2^, partial eta-squared effect size.*

### Cognitive Tests

#### Interaction Effect

Significant interaction effects between *Time* and *Group* were detected for DSC. The TBI-MF group did not improve their speed during the second DSC test, whereas the controls became faster (Figure 3K). No other *Time* and *Group* interaction was found for the other tests (Table 2). There was no interaction for the *Stroop* and *Group* [*F*(1, 37) = 4, 725; FDR adjusted *p* > 0.05, η_p_^2^ = 0.113]. Nor were any *Simon* and *Group* interactions found [*F*(1, 37) = 0.003; FDR adjusted *p* > 0.05, η_p_^2^ < 0.001].

#### Group Effect

The TBI-MF group was significantly slower than the control group on SS, PaSMO, SAWM, Stroop–Simon, and DSC (Table 2 and Figure 3B,F,H,J,L). The additional *post hoc t*-test for the DSC did not show any difference between the groups during the first test session (*t* = −1.859, FDR adjusted *p* > 0.05, with a Cohen’s *d* of 0.57), while the TBI-MF was significantly slower in the second test session (*t* = −2.988, FDR adjusted *p* < 0.05, with a Cohen’s *d* of 0.86). No differences in reaction time nor errors made were found between the groups for the OPATUS-CPTA, but the rate of omissions was significantly higher in the TBI-MF group, as compared to controls (*t* = 2.472, FDR adjusted *p* < 0.05). No difference was found between groups with respect to Digit Span, and errors made in any of the tests (SAWM, PaSMO, and Stroop–Simon). For comparison, data adjusted to age according to WAIS-IV manual, DSC, SS, and DS were within the normal range for both groups.

### fNIRS Result

#### Stroop–Simon

**FIGURE 4:**
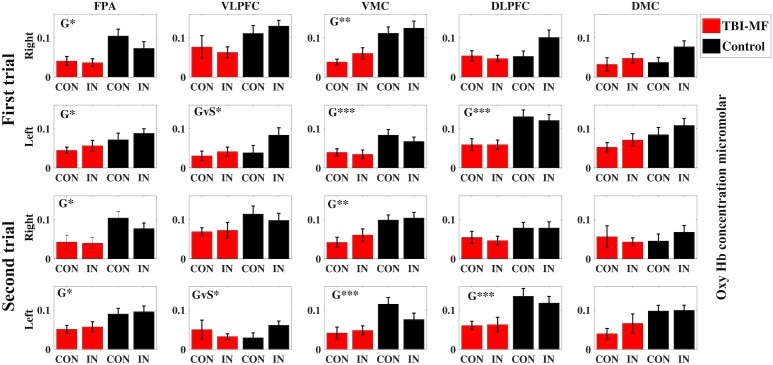
Concentration of oxygenated hemoglobin (micromolar) during fNIRS imaging of the Stroop–Simon test separated by test sessions (First and Second) and hemispheres (Right and Left) for all regions of interest (FPA, frontal polar area; VLPFC, ventral lateral prefrontal cortex; VMC, ventral motor cortex; DLPFC, dorsolateral prefrontal cortex; DMC, dorsal motor cortex). The red columns indicate data for the TBI-MF group, the black columns for healthy controls. *CON* refers to Stroop congruent trials, and *IN* refers to Stroop incongruent trials, as described in the Materials and Methods section. The error bars indicate standard error of mean. Significant effects are indicated for Group (G) or Group-Stroop interaction (GvS). ^∗∗∗^FDR adjusted *p* < 0.001, ^∗∗^FDR adjusted *p* < 0.01, ^∗^FDR adjusted *p* < 0.05.

No *Group* and *Time* interactions were found for any of the ROIs (Figure 4 and Table 3). There was a *Group* effect in bilateral FPA, bilateral VMC and left DLPFC, with the TBI-MF group having lower concentrations of oxy-Hb compared to controls in the mentioned ROIs (see Figure 4 and Supplementary Figures S1–S4 for the hemodynamic response curves for each ROI and for single channels). A *Stroop* and *Group* interaction was found in the left VLPFC showing that the TBI-MF group did have the same oxy-Hb concentration for both congruent and incongruent trials, whereas the controls had more oxy-Hb in the incongruent trial, compared to the congruent trial. To explore the Stroop effect (the oxy-Hb difference between incongruent and congruent trials) in the left VLPFC, we conducted a Pearson’s correlation between the Stroop effect and the first VAS, the second VAS, the difference between the first and second VAS as well as MFS. None of the correlation with VAS was significant with the first VAS (*r* = −0.04, FDR adjusted *p* > 0.05), the second VAS (*r* = −0.282, FDR adjusted *p* > 0.05) and the VAS difference (*r* = −0.234, FDR adjusted *p* > 0.05). The correlation with MFS was found to be significant (*r* = −0.399, FDR adjusted *p* < 0.05), showing that higher MFS scores were associated with lower oxy-Hb concentrations (see Supplementary Figure S5).

**Table 3:** Repeated ANOVA for the Stroop fNIRS data.

		Group		Time vs. Group			Stroop Effect vs. Group		
	F	FDR	η_p_^2^	F	FDR	η_p_^2^	F	FDR	η_p_^2^
rFPA	9.342	^∗^	0.202	0.002	ns	0.000	5.303	ns	0.125
lFPA	5.630	^∗^	0.132	0.455	ns	0.012	0.026	ns	0.001
rVLPFC	4.030	ns	0.098	0.525	ns	0.014	0.125	ns	0.003
lVLPFC	1.112	ns	0.029	0.612	ns	0.016	10.876	^∗^	0.227
rVMC	12.761	^∗∗^	0.256	1.152	ns	0.030	1.391	ns	0.036
lVMC	15.588	^∗∗∗^	0.296	0.211	ns	0.006	7.218	ns	0.163
rDLPFC	4.632	ns	0.111	0.005	ns	0.000	5.199	ns	0.123
lDLPFC	19.619	^∗∗∗^	0.347	0.005	ns	0.000	0.558	ns	0.015
rDMC	0.552	ns	0.015	0.174	ns	0.005	4.437	ns	0.107
lDMC	4.912	ns	0.117	0.260	ns	0.007	0.620	ns	0.016

*FDR, p-value after adjustment with false discovery rate, ^∗∗∗^p < 0.001, ^∗∗^p < 0.01, ^∗^p < 0.05, ^ns^p > 0.05; η_p_^2^, partial eta-squared effect size; r, right; l, left; FPA, frontal polar area; VLPFC, ventral lateral prefrontal cortex; VMC, ventral motor cortex; DLPFC, dorsolateral prefrontal cortex; DMC, dorsal motor cortex.*

## Discussion

The present study examined how prolonged mental activity affects cognitive performance and its neural correlates in individuals with TBI-MF. The TBI-MF group demonstrated lower event-related activity in the FPA, VLPFC, and DLPFC, compared to controls. Group difference in brain activity was detected already during the first test session. No difference in brain activity between the two test sessions was found, and this was the case for both groups.

The TBI-MF participants had difficulty utilizing the left VLPFC in the conflict processing, with no difference in oxy-Hb between congruent and incongruent trials. This was not the case for the controls, who had higher levels of oxy-Hb for the incongruent than congruent trials, indicating a capacity to recruit more activation when the task was more demanding. The left VLPFC, involving inferior frontal gyrus, is known to be involved in semantic conflict processing in Stroop (Egner and Hirsch, 2005; Yasumura et al., 2014; Musz and Thompson-Schill, 2017). A correlation between increased difficulties in utilizing the left VLPFC for the semantic conflict and higher rating on the MFS, was detected. This correlation should, however, be interpreted with caution since a score above 10.5 was an inclusion criterion for the TBI-MF group. This is in line with previous findings by Berginstrom and colleagues, where a modified version of Symbol Digit Modality Test (SDMT) was used, and a correlation between self-assessed fatigue and decreased brain activity in the right inferior and middle frontal gyrus was reported (Berginstrom et al., 2017). We also hypothesized that we would detect this difference in the DLPFC, an area important for cognitive control (Vanderhasselt et al., 2009). We found a difference as indicated by the high *F*-value for the interaction between group and the Stroop effect in the right DLPFC (see Table 3), but it did not reach statistical significance after correcting for multiple tests.

To our knowledge, this is the first fNIRS study focusing on MF after mild TBI. There is one previous study by Plenger et al. that used fNIRS while performing a Stroop test in a cohort of individuals with moderate to severe TBI (Plenger et al., 2016). Their results suggest impaired frontal cortex conflict processing indicated by no difference in oxy-Hb concentration levels between the simple dot-color naming task and the more demanding incongruent task. Several other fNIRS studies with moderate to severer TBI have reported a reduced brain activity for a variety of cognitive tasks such as visual discrimination, attention and working memory, especially in DLPFC (Merzagora et al., 2011; Hibino et al., 2013; Kontos et al., 2014; Sawamura et al., 2014; Helmich et al., 2015) but also in VLPFC (Hibino et al., 2013). However, fatigue was not assessed in these fNIRS studies, and, in contrast to our sample, they studied individuals with moderate to severe TBI, whereas we included individuals with mild TBI in our study. A recent fNIRS study, including participants with post-concussion symptoms (PCS) after a mild TBI, showed reduced connectivity for the mild TBI group compared to controls and a correlation between reduced coherence and increased symptom severity (Hocke et al., 2018). In their study, fatigue was not reported, despite the fact that fatigue is one of the symptoms included in the PCS.

In previous fMRI studies, changes in brain activity have been reported to be related to fatigue. During the performance of a Symbol Digit Modalities Test task, Kohl et al. (2009) found increased brain activity during the 30 min test period among participants who had suffered a moderate to severe TBI. In contrast, a decreased activity was reported for the controls in several brain regions, including middle frontal gyrus, superior parietal cortex, basal ganglia and anterior cingulate (Kohl et al., 2009). Another study using the same task reported decreased brain activity in deeper brain structures, in particular in the basal ganglia, primarily caudate nucleus, the thalamus and anterior insula in the TBI group (mild, moderate, severe) as compared to controls (Berginstrom et al., 2017). The activity of the controls decreased across the 27 min test session, whereas the TBI participants remained on a similar lower activity level (Berginstrom et al., 2017). A recent fMRI study measuring fatigue among individuals who had suffered a moderate to severe TBI, found an interaction between brain activity and cognitive tasks, in the tail of the caudate nucleus (Wylie et al., 2017). Fatigue after mild TBI also correlated with abnormal functional connectivity in the thalamus and middle frontal cortex (Nordin et al., 2016). Altered cerebral blood flow in mild TBI was also reported, mainly in the frontal cortex and thalamic networks (Moller et al., 2017).

A difference was found in the DSC task of processing speed with the controls improving their performance during the second test session, whereas the TBI-MF group did not. Similarly, improved performance over time for controls has been reported, with a better performance over time, and with no change in speed for TBI patients (Ashman et al., 2008; Johansson and Ronnback, 2015; Jonasson et al., 2018). This indicated a fatiguing effect with reduced efficiency for those suffering from mental fatigue during a longer cognitive activity. However, the relationship between effectiveness and efficiency needs to be interpreted, e.g., a low effectiveness (more errors) may be due to a high efficiency (fast response time). In this study, the controls and TBI-MF did not differ in their effectiveness, which was measured by error rate in the cognitive tests. In contrast, a difference was reported in efficiency, measured in terms of response time, with controls improving during the second test session, while the TBI-MF participants remained at a similar level as during the first test session.

Regarding performance in cognitive tests, the TBI-MF group was slower than the control group on SS, PaSMO, SAWM, DSC and Simon-Stoop and made more omissions on OPATUS-CPTA, while no difference in working memory (DS) was found. Related to the omission of the OPATUS-CPTA, there was a high rate of omissions in the Stroop–Simon test for the TBI-MF group as indicated by the high *F*-value (see Table 2). It did, however, not reach statistical significance after correcting for multiple tests. The higher rate of omissions is an indicator of the problem of keeping a sustained attention for the TBI-MF group. The performance on the WAIS-IV tests included in this study (SS, DSC, DS) was within normal range for all participants, even though the TBI-MF group suffered from MF, as indicated by their rating on the MFS. Differences in cognitive performance at a group level have been reported previously and it has been proposed that cognitive decline is related to fatigue among people who had suffered a mild TBI (Azouvi et al., 2004; Ziino and Ponsford, 2006a; Ashman et al., 2008; Belmont et al., 2009; Johansson et al., 2009). The TBI-MF group was less efficient, and the participants’ decreased experience of mental energy from start to the end of the test session suggest an objective and subjective fatigability due to the mental load they were exposed for during 2½ h.

The difference in brain activity between the groups was reported during the first test session using the VAS scale, but no differences were reported as to how they experienced their energy level at start of the test procedure. This may have implications for the ability to adapt to a balanced activity level in daily living for the TBI-MF group. From years of clinical experience, most people suffering from TBI-MF report that finding a sustainable daily activity level is challenging. They rest when they feel fatigued, which is natural, but it is difficult to learn to take rests more regularly during the day if they do not feel the need to do so. Many subjects also report about overdoing activities when they temporarily feel “normal,” but they later suffer from severe exhaustion. This finding also fits in with the discrepancy between subjective experience and actual brain function.

## Limitations

The Stroop–Simon test adopted for fNIRS had an average stimulus-response time of 9 s in this study. Because the hemodynamic response normally takes 12 s to return to baseline, a shorter time interval could result in the oxy-Hb not returning to baseline. Since change in the oxy-Hb and deoxy-Hb concentration is calculated from a relative baseline, this shorter interval could possibly affect the sensitivity of oxy-Hb as a measure of brain activity. However, no additive increase of oxy-Hb was found, implying that the lower frontal cortex activity for the TBI-MF group cannot be explained by their hemodynamic concentration levels not returning to baseline after 9 s (see the Supplementary Material, Supplementary Figure S6). A longer stimulus-response time would have enabled us to also measure other properties of the hemodynamic response, e.g., time-to-peak or time-to-baseline. It was not possible to analyze fNIRS Simon effect due to coding problems. Due to the design of our Stroop–Simon test, we are also lacking hemodynamic response data for easier trials as used by Plenger et al. (2016), where naming a color dot represents an easier task as compared to our study using congruent and incongruent stimulus-response pairs. It is possible that the long recording of the Stroop–Simon task could have induced artifacts to the data. However, since we presume this to create random error in the data and the main question related to between-group differences, this should not affect the interpretation of the data.

Additional analysis with the deoxy-Hb could have yielded complementary information. However, the wavelength of the fNIRS system used was better suited for oxy-Hb, and since we did not find any additional information from the basic analysis of deoxy-Hb, we chose to exclusively analyze oxy-Hb. Further analysis would also have led to more corrections which, in turn could have led to more type II errors.

Correction for multiple testing could have obscured true differences, i.e., a type II error. However, *t*- and *F*-values, degrees of freedom and effect sizes are reported here for assessing results. Because we did not have a second control group with mild TBI without MF we are not able to discriminate between effects of mental fatigue and the effects of mild TBI. Since we focused on studying conflict processing, processing speed, attention, working memory and mental control, we did not include additional measurements of learning or memory. Therefore, we cannot discriminate to what degree the deterioration in the performance on the DSC for the TBI-MF is due to an associated memory problem.

## Conclusion

We found indications that individuals with TBI-MF have a reduced efficiency of neuronal activity in the frontal cortex. This may result both in deterioration of cognitive function and the experience of a mental fatigue after extended mental activity.

## Ethics Statement

This study was carried out in accordance with the recommendations of Ethical Review Board in Gothenburg with written informed consent from all subjects. All subjects gave written informed consent in accordance with the Declaration of Helsinki. The protocol was approved by the Ethical Review Board in Gothenburg.

## Author Contributions

BJ and SS conceived, planned, and carried out the experiments. BJ, HK, and SS analyzed the data. BJ and SS took the lead in writing the manuscript. All authors made significant contribution to the writing of the manuscript.

## Conflict of Interest Statement

The authors declare that the research was conducted in the absence of any commercial or financial relationships that could be construed as a potential conflict of interest.

## Supplementary Material

FIGURE S1 The oxy-Hb and deoxy-Hb concentration for the incongruent trials in the Stroop-Simon test for all five regions of interest in each hemisphere (FPA – frontal polar area, VLPFC – ventral lateral prefrontal cortex, VMC – ventral motor cortex, DLPFC – dorsolateral prefrontal cortex, DMC – dorsal motor cortex). fNIRS activity for the TBI-MF group is depicted with solid lines and controls with dashed lines. The oxy-Hb concentration in indicated in red and the deoxy-Hb in blue. All y-axis are presented as μ molar concentrations of oxy-Hb and deoxy-Hb. Significant effects are indicated for Group (G) or Group-Stroop interaction (GvS). ^∗∗∗^FDR adjusted *p* < 0.001, ^∗∗^FDR adjusted *p* < 0.01, ^∗^FDR adjusted *p* < 0.05.

FIGURE S2 fNIRS channel locations viewed from above.

FIGURE S3 Oxy-Hb and deoxy-Hb concentration with short separation regression for Stroop incongruent trials in the Stroop-Simon test for one channel from each of the ten regions of interest. The TBI-MF group activity is depicted with the solid lines and the dashed lines represent the controls. Red lines indicate oxy-Hb concentrations and blue lines deoxy-Hb concentrations.

FIGURE S4 Oxy-Hb and deoxy-Hb concentration without short separation regression for Stroop incongruent trials in the Stroop-Simon test for one channel from each of the ten regions of interest. The TBI-MF group activity is depicted with the solid lines and the dashed lines represent the controls. Red lines indicate oxy-Hb concentrations and blue lines deoxy-Hb concentrations.

FIGURE S5 Pearson’s correlation between the mental fatigue scale (MFS) and Stroop effect in the left VLPFC. The Stroop effect is calculated in the fNIRS data as the oxy-Hb difference between incongruent and congruent trials in the Stroop dimension. **(A)** Correlation of *r* = −0.399, a possible outlier is highlighted with a rectangle. **(B)** Correlation of *r* = −0.442, the association is still present with the potential outlier removed. Both correlations are statistically significant with an FDR adjusted *p* < 0.05.

FIGURE S6 The oxy-Hb concentration for the complete first Stroop-Simon test session (23 min) for all ten ROI. The black curves indicate controls and the red curves the TBI-MF group. If the smaller oxy-Hb response of TBI-MF group were due to a maximum ceiling effect, we would predict a steady increase for the red curves. Since we did not observe such increase, we propose that the shortened stimulus-stimulus interval of 7-11 seconds had not affected the oxy-Hb levels of the TBI-MF group more than the controls.

## References

[B1] Ahman S. Saveman B. I. Styrke J. Bjornstig U. Stalnacke B. M. (2013). Long-term follow-up of patients with mild traumatic brain injury: a mixed-method study. *J. Rehabil. Med.* 45 758–764. 10.2340/16501977-1182 24002311

[B2] Ashman T. A. Cantor J. B. Gordon W. A. Spielman L. Egan M. Ginsberg A. (2008). Objective measurement of fatigue following traumatic brain injury. *J. Head. Trauma Rehabil.* 23 33–40. 10.1097/01.htr.0000308719.70288.22 18219233

[B3] Azouvi P. Couillet J. Leclercq M. Martin Y. Asloun S. Rousseaux M. (2004). Divided attention and mental effort after severe traumatic brain injury. *Neuropsychologia* 42 1260–1268. 10.1016/j.neuropsychologia.2004.01.001 15178177

[B4] Balardin J. B. Zimeo Morais G. A. Furucho R. A. Trambaiolli L. Vanzella P. Biazoli C. (2017). Imaging brain function with functional near-infrared spectroscopy in unconstrained environments. *Front. Hum. Neurosci.* 11:258. 10.3389/fnhum.2017.00258 28567011 PMC5434677

[B5] Belmont A. Agar N. Azouvi P. (2009). Subjective fatigue, mental effort, and attention deficits after severe traumatic brain injury. *Neurorehabil. Neural Repair* 23 939–944. 10.1177/1545968309340327 19574545

[B6] Berginstrom N. Nordstrom P. Ekman U. Eriksson J. Andersson M. Nyberg L. (2017). Using functional magnetic resonance imaging to detect chronic fatigue in patients with previous traumatic brain injury: changes linked to altered striato-thalamic-cortical functioning. *J. Head. Trauma Rehabil.* 33 266–274. 10.1097/htr.0000000000000340 28926483

[B7] Borragán G. Gilson M. Atas A. Slama H. Lysandropoulos A. De Schepper M. (2018). Cognitive fatigue, sleep and cortical activity in multiple sclerosis disease. a behavioral, polysomnographic and functional near-infrared spectroscopy investigation. *Front. Hum. Neurosci.* 12:378. 10.3389/fnhum.2018.00378 30294266 PMC6158319

[B8] Brigadoi S. Cooper R. J. (2015). How short is short? Optimum source-detector distance for short-separation channels in functional near-infrared spectroscopy. *Neurophotonics* 2:025005. 10.1117/1.NPh.2.2.025005 26158009 PMC4478880

[B9] Byun K. Hyodo K. Suwabe K. Ochi G. Sakairi Y. Kato M. (2014). Positive effect of acute mild exercise on executive function via arousal-related prefrontal activations: an fNIRS study. *Neuroimage* 98 336–345. 10.1016/j.neuroimage.2014.04.067 24799137

[B10] Cantor J. B. Ashman T. Gordon W. Ginsberg A. Engmann C. Egan M. (2008). Fatigue after traumatic brain injury and its impact on participation and quality of life. *J. Head. Trauma Rehabil.* 23 41–51. 10.1097/01.HTR.0000308720.70288.af 18219234

[B11] Carroll L. J. Cassidy J. D. Peloso P. M. Borg J. von Holst H. Holm L. (2004). Prognosis for mild traumatic brain injury: results of the WHO collaborating centre task force on mild traumatic brain injury. *J. Rehabil. Med.* 43(Suppl.), 84–105. 10.1080/16501960410023859 15083873

[B12] Chaudhuri A. Behan P. O. (2004). Fatigue in neurological disorders. *Lancet* 363 978–988. 10.1016/s0140-6736(04)15794-2 15043967

[B13] Dux P. E. Marois R. (2009). The attentional blink: a review of data and theory. *Atten. Percept. Psychophys.* 71 1683–1700. 10.3758/app.71.8.1683 19933555 PMC2915904

[B14] Egner T. (2008). Multiple conflict-driven control mechanisms in the human brain. *Trends Cogn. Sci.* 12 374–380. 10.1016/j.tics.2008.07.001 18760657

[B15] Egner T. Delano M. Hirsch J. (2007). Separate conflict-specific cognitive control mechanisms in the human brain. *Neuroimage* 35 940–948. 10.1016/j.neuroimage.2006.11.061 17276088

[B16] Egner T. Hirsch J. (2005). The neural correlates and functional integration of cognitive control in a Stroop task. *Neuroimage* 24 539–547. 10.1016/j.neuroimage.2004.09.007 15627596

[B17] Ehlis A. C. Schneider S. Dresler T. Fallgatter A. J. (2014). Application of functional near-infrared spectroscopy in psychiatry. *Neuroimage* 85 478–488. 10.1016/j.neuroimage.2013.03.067 23578578

[B18] Endo K. Matsukawa K. Liang N. Nakatsuka C. Tsuchimochi H. Okamura H. (2013). Dynamic exercise improves cognitive function in association with increased prefrontal oxygenation. *J. Physiol. Sci.* 63 287–298. 10.1007/s12576-013-0267-6 23661275 PMC10717244

[B19] Everdell N. L. Gibson A. P. Tullis I. D. C. Vaithianathan T. Hebden J. C. Delpy D. T. (2005). A frequency multiplexed near-infrared topography system for imaging functional activation in the brain. *Rev. Sci. Instru.* 76:093705 10.1063/1.2038567

[B20] Ferrari M. Quaresima V. (2012). A brief review on the history of human functional near-infrared spectroscopy (fNIRS) development and fields of application. *Neuroimage* 63 921–935. 10.1016/j.neuroimage.2012.03.049 22510258

[B21] Forster S. E. Cho R. Y. (2014). Context specificity of post-error and post-conflict cognitive control adjustments. *PLoS One* 9:e90281. 10.1371/journal.pone.0090281 24603900 PMC3946012

[B22] Frencham K. A. Fox A. M. Maybery M. T. (2005). Neuropsychological studies of mild traumatic brain injury: a meta-analytic review of research since 1995. *J. Clin. Exp. Neuropsychol.* 27 334–351. 10.1080/13803390490520328 15969356

[B23] Gagnon L. Perdue K. Greve D. N. Goldenholz D. Kaskhedikar G. Boas D. A. (2011). Improved recovery of the hemodynamic response in diffuse optical imaging using short optode separations and state-space modeling. *Neuroimage* 56 1362–1371. 10.1016/j.neuroimage.2011.03.001 21385616 PMC3085546

[B24] Haber S. N. Calzavara R. (2009). The cortico-basal ganglia integrative network: the role of the thalamus. *Brain Res. Bull.* 78 69–74. 10.1016/j.brainresbull.2008.09.013 18950692 PMC4459637

[B25] Hawthorne G. Gruen R. L. Kaye A. H. (2009). Traumatic brain injury and long-term quality of life: findings from an Australian study. *J. Neurotrauma* 26 1623–1633. 10.1089/neu.2008-073510.1089/neu.2008.0735 19317590

[B26] Helmich I. Saluja R. S. Lausberg H. Kempe M. Furley P. Berger A. (2015). Persistent postconcussive symptoms are accompanied by decreased functional brain oxygenation. *J. Neuropsychiatr. Clin. Neurosci.* 27 287–298. 10.1176/appi.neuropsych.14100276 25803447

[B27] Hibino S. Mase M. Shirataki T. Nagano Y. Fukagawa K. Abe A. (2013). Oxyhemoglobin changes during cognitive rehabilitation after traumatic brain injury using near infrared spectroscopy. *Neurol. Med. Chir.* 53 299–303. 23708220 10.2176/nmc.53.299

[B28] Hocke L. M. Duszynski C. C. Debert C. T. Dleikan D. Dunn J. F. (2018). Reduced functional connectivity in adults with persistent post-concussion symptoms: a functional near-infrared spectroscopy study. *J. Neurotrauma* 35 1224–1232. 10.1089/neu.2017.5365 29373947 PMC5962910

[B29] Huppert T. J. Diamond S. G. Franceschini M. A. Boas D. A. (2009). HomER: a review of time-series analysis methods for near-infrared spectroscopy of the brain. *Appl. Opt.* 48 D280–D298. 19340120 10.1364/ao.48.00d280 PMC2761652

[B30] Hyodo K. Dan I. Suwabe K. Kyutoku Y. Yamada Y. Akahori M. (2012). Acute moderate exercise enhances compensatory brain activation in older adults. *Neurobiol. Aging* 33 2621–2632. 10.1016/j.neurobiolaging.2011.12.022 22300952

[B31] Irani F. Platek S. M. Bunce S. Ruocco A. C. Chute D. (2007). Functional near infrared spectroscopy (fNIRS): an emerging neuroimaging technology with important applications for the study of brain disorders. *Clin. Neuropsychol.* 21 9–37. 10.1080/13854040600910018 17366276

[B32] Jo Bsis-Vandervliet F. F. (1999). Discovery of the near-infrared window into the body and the early development of near-infrared spectroscopy. *J. Biomed. Opt.* 4 392–396. 10.1117/1.429952 23014610

[B33] Johansson B. Berglund P. Ronnback L. (2009). Mental fatigue and impaired information processing after mild and moderate traumatic brain injury. *Brain Inj.* 23 1027–1040. 10.3109/02699050903421099 19909051

[B34] Johansson B. Ronnback L. (2014). Evaluation of the mental fatigue scale and its relation to cognitive and emotional functioning after traumatic brain injury or stroke. *Int. J. Phys. Med. Rehabil.* 2 572–573. 10.4172/2329-9096.1000182

[B35] Johansson B. Rönnbäck L. (2014). “Long-lasting mental fatigue after traumatic brain injury – a major problem most often neglected diagnostic criteria, assessment, relation to emotional and cognitive problems, cellular background, and aspects on treatment,” in *Traumatic Brain Injury*, ed. Sadaka F. (Rijeka: InTech).

[B36] Johansson B. Ronnback L. (2015). Novel computer tests for identification of mental fatigue after traumatic brain injury. *Neurorehabilitation* 36 195–202. 10.3233/nre-151207 25882202

[B37] Johansson B. Starmark A. Berglund P. Rodholm M. Ronnback L. (2010). A self-assessment questionnaire for mental fatigue and related symptoms after neurological disorders and injuries. *Brain Inj.* 24 2–12. 10.3109/02699050903452961 20001478

[B38] Jonasson A. Levin C. Renfors M. Strandberg S. Johansson B. (2018). Mental fatigue and impaired cognitive function after an acquired brain injury. *Brain Behav.* 8:e01056. 10.1002/brb3.1056 29956894 PMC6085903

[B39] King N. S. Crawford S. Wenden F. J. Moss N. E. Wade D. T. (1995). The Rivermead post concussion symptoms questionnaire: a measure of symptoms commonly experienced after head injury and its reliability. *J. Neurol.* 242 587–592. 8551320 10.1007/BF00868811

[B40] Kohl A. D. Wylie G. R. Genova H. M. Hillary F. G. Deluca J. (2009). The neural correlates of cognitive fatigue in traumatic brain injury using functional MRI. *Brain Inj.* 23 420–432. 10.1080/02699050902788519 19408165

[B41] Kontos A. P. Huppert T. J. Beluk N. H. Elbin R. J. Henry L. C. French J. (2014). Brain activation during neurocognitive testing using functional near-infrared spectroscopy in patients following concussion compared to healthy controls. *Brain Imaging Behav.* 8 621–634. 10.1007/s11682-014-9289-9 24477579 PMC5080617

[B42] Kopton I. M. Kenning P. (2014). Near-infrared spectroscopy (NIRS) as a new tool for neuroeconomic research. *Front. Hum. Neurosci.* 8:549. 10.3389/fnhum.2014.00549 25147517 PMC4124877

[B43] Kraus J. F. Hsu P. Schafer K. Afifi A. A. (2014). Sustained outcomes following mild traumatic brain injury: results of a five-emergency department longitudinal study. *Brain Inj.* 28 1248–1256. 10.3109/02699052.2014.916420 24841806

[B44] Lague-Beauvais M. Brunet J. Gagnon L. Lesage F. Bherer L. (2013). A fNIRS investigation of switching and inhibition during the modified Stroop task in younger and older adults. *Neuroimage* 64 485–495. 10.1016/j.neuroimage.2012.09.042 23000257

[B45] Lannsjo M. Af Geijerstam J. L. Johansson U. Bring J. Borg J. (2009). Prevalence and structure of symptoms at 3 months after mild traumatic brain injury in a national cohort. *Brain Inj.* 23 213–219. 10.1080/02699050902748356 19205957

[B46] Leon-Carrion J. Damas-Lopez J. Martin-Rodriguez J. F. Dominguez-Roldan J. M. Murillo-Cabezas F. Barroso Y. M. J. M. (2008). The hemodynamics of cognitive control: the level of concentration of oxygenated hemoglobin in the superior prefrontal cortex varies as a function of performance in a modified Stroop task. *Behav. Brain Res.* 193 248–256. 10.1016/j.bbr.2008.06.013 18606191

[B47] Lindqvist G. Malmgren H. (1993). Organic mental disorders as hypothetical pathogenetic processes. *Acta Psychiatr. Scand. Suppl.* 373 5–17. 8372702 10.1111/j.1600-0447.1993.tb05611.x

[B48] MATLAB (2014). *MATLAB.* Massachusetts, MA: The MathWorks, Inc.

[B49] McInnes K. Friesen C. L. MacKenzie D. E. Westwood D. A. Boe S. G. (2017). Mild traumatic brain injury (mTBI) and chronic cognitive impairment: a scoping review. *PLoS One* 12:e0174847. 10.1371/journal.pone.0174847 28399158 PMC5388340

[B50] Merzagora A. C. Schultheis M. T. Onaral B. Izzetoglu M. (2011). Functional near-infrared spectroscopy-based assessment of attention impairments after traumatic brain injury. *J. Innovat. Opt. Health Sci.* 4 251–260. 10.1142/S1793545811001551

[B51] Moller M. C. Nordin L. E. Bartfai A. Julin P. Li T. Q. (2017). Fatigue and cognitive fatigability in mild traumatic brain injury are correlated with altered neural activity during vigilance test performance. *Front. Neurol.* 8:496. 10.3389/fneur.2017.00496 28983280 PMC5613211

[B52] Musz E. Thompson-Schill S. L. (2017). Tracking competition and cognitive control during language comprehension with multi-voxel pattern analysis. *Brain Lang.* 165 21–32. 10.1016/j.bandl.2016.11.002 27898341 PMC5359984

[B53] Nguyen R. Fiest K. M. McChesney J. Kwon C. S. Jette N. Frolkis A. D. (2016). The international incidence of traumatic brain injury: a systematic review and meta-analysis. *Can. J. Neurol. Sci.* 43 774–785. 10.1017/cjn.2016.290 27670907

[B54] Nordin L. E. Moller M. C. Julin P. Bartfai A. Hashim F. Li T. Q. (2016). Post mTBI fatigue is associated with abnormal brain functional connectivity. *Sci. Rep.* 6:21183. 10.1038/srep21183 26878885 PMC4754765

[B55] Obrig H. (2014). NIRS in clinical neurology - a ‘promising’ tool? *Neuroimage* 85(Pt 1), 535–546. 10.1016/j.neuroimage.2013.03.045 23558099

[B56] Park N. W. Moscovitch M. Robertson I. H. (1999). Divided attention impairments after traumatic brain injury. *Neuropsychologia* 37 1119–1133. 10509834 10.1016/s0028-3932(99)00034-2

[B57] Plenger P. Krishnan K. Cloud M. Bosworth C. Qualls D. Marquez de la Plata C. (2016). fNIRS-based investigation of the Stroop task after TBI. *Brain Imaging Behav.* 10 357–366. 10.1007/s11682-015-9401-9 26058665

[B58] Ponsford J. Cameron P. Fitzgerald M. Grant M. Mikocka-Walus A. (2011). Long-term outcomes after uncomplicated mild traumatic brain injury: a comparison with trauma controls. *J. Neurotrauma* 28 937–946. 10.1089/neu.2010.1516 21410321

[B59] Reitan R. M. Wolfson D. (1985). *The Halstead-Reitan Neuropsychological test Battery: Theory and Clinical Interpretation.* Tucson: Neuropsychology Press.

[B60] Roberts K. L. Hall D. A. (2008). Examining a supramodal network for conflict processing: a systematic review and novel functional magnetic resonance imaging data for related visual and auditory stroop tasks. *J. Cogn. Neurosci.* 20 1063–1078. 10.1162/jocn.2008.20074 18211237

[B61] Rodriguez Merzagora A. C. Izzetoglu M. Onaral B. Schultheis M. T. (2014). Verbal working memory impairments following traumatic brain injury: an fNIRS investigation. *Brain Imaging Behav.* 8 446–459. 10.1007/s11682-013-9258-8 24085609

[B62] Sato H. Yahata N. Funane T. Takizawa R. Katura T. Atsumori H. (2013). A NIRS-fMRI investigation of prefrontal cortex activity during a working memory task. *Neuroimage* 83 158–173. 10.1016/j.neuroimage.2013.06.043 23792984

[B63] Sawamura D. Ikoma K. Yoshida K. Inagaki Y. Ogawa K. Sakai S. (2014). Active inhibition of task-irrelevant sounds and its neural basis in patients with attention deficits after traumatic brain injury. *Brain Inj.* 28 1455–1460. 10.3109/02699052.2014.919531 24946201

[B64] Schroeter M. L. Zysset S. Kupka T. Kruggel F. Yves von Cramon D. (2002). Near-infrared spectroscopy can detect brain activity during a color-word matching Stroop task in an event-related design. *Hum. Brain Mapp.* 17 61–71. 10.1002/hbm.10052 12203689 PMC6872032

[B65] Schroeter M. L. Zysset S. Wahl M. von Cramon D. Y. (2004). Prefrontal activation due to Stroop interference increases during development–an event-related fNIRS study. *Neuroimage* 23 1317–1325. 10.1016/j.neuroimage.2004.08.001 15589096

[B66] Singh A. K. Dan I. (2006). Exploring the false discovery rate in multichannel NIRS. *Neuroimage* 33 542–549. 10.1016/j.neuroimage.2006.06.047 16959498

[B67] Singh A. K. Okamoto M. Dan H. Jurcak V. Dan I. (2005). Spatial registration of multichannel multi-subject fNIRS data to MNI space without MRI. *Neuroimage* 27 842–851. 10.1016/j.neuroimage.2005.05.019 15979346

[B68] Uludag K. Steinbrink J. Villringer A. Obrig H. (2004). Separability and cross talk: optimizing dual wavelength combinations for near-infrared spectroscopy of the adult head. *Neuroimage* 22 583–589. 10.1016/j.neuroimage.2004.02.023 15193586

[B69] Vanderhasselt M. A. De Raedt R. Baeken C. (2009). Dorsolateral prefrontal cortex and Stroop performance: tackling the lateralization. *Psychon. Bull. Rev.* 16 609–612. 10.3758/pbr.16.3.609 19451392

[B70] Wechsler D. (2010). *Wechsler Adut Intelligence Scale - Fourth Edition, Swedish Edition.* Stockholm: Pearson Assessment.

[B71] Wylie G. R. Dobryakova E. DeLuca J. Chiaravalloti N. Essad K. Genova H. (2017). Cognitive fatigue in individuals with traumatic brain injury is associated with caudate activation. *Sci. Rep.* 7:8973. 10.1038/s41598-017-08846-6 28827779 PMC5567054

[B72] Yasumura A. Kokubo N. Yamamoto H. Yasumura Y. Nakagawa E. Kaga M. (2014). Neurobehavioral and hemodynamic evaluation of Stroop and reverse Stroop interference in children with attention-deficit/hyperactivity disorder. *Brain Dev.* 36 97–106. 10.1016/j.braindev.2013.01.005 23414618

[B73] Ye J. C. Tak S. Jang K. E. Jung J. Jang J. (2009). NIRS-SPM: statistical parametric mapping for near-infrared spectroscopy. *Neuroimage* 44 428–447. 10.1016/j.neuroimage.2008.08.036 18848897

[B74] Ziino C. Ponsford J. (2006a). Selective attention deficits and subjective fatigue following traumatic brain injury. *Neuropsychology* 20 383–390. 10.1037/0894-4105.20.3.383 16719631

[B75] Ziino C. Ponsford J. (2006b). Vigilance and fatigue following traumatic brain injury. *J. Int. Neuropsychol. Soc.* 12 100–110. 10.1017/s1355617706060139 16433949

