# Supplementary figures and images for: Mental Fatigue and Functional Near-Infrared Spectroscopy (fNIRS) – Based Assessment of Cognitive Performance After Mild Traumatic Brain Injury
Source: Front Hum Neurosci. 2019 May 14;13:145. doi: 10.3389/fnhum.2019.00145 (PMC6527600; doi:10.3389/fnhum.2019.00145)

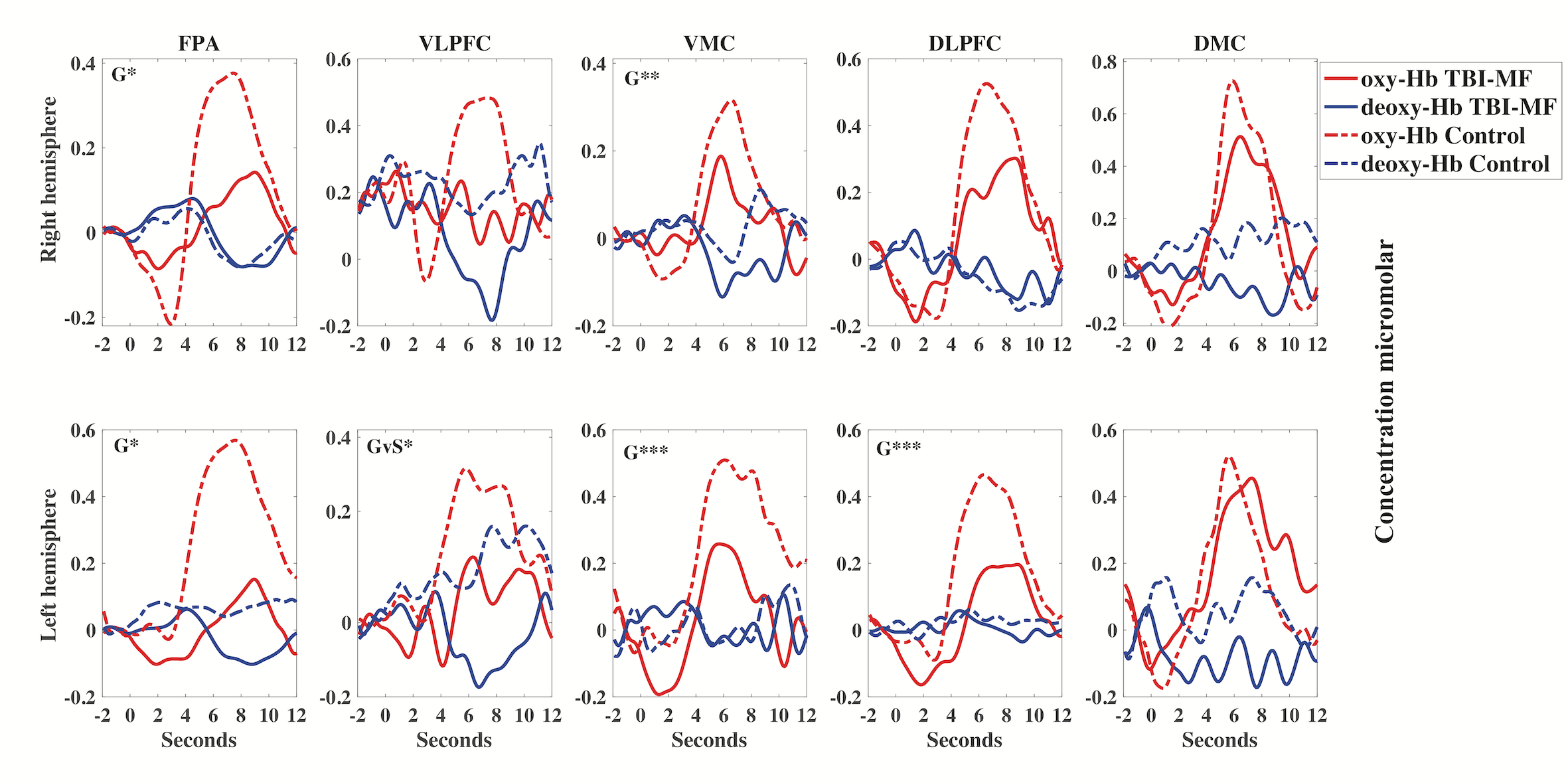

Supplement: FIGURE S1 — The oxy-Hb and deoxy-Hb concentration for the incongruent trials in the Stroop-Simon test for all five regions of interest in each hemisphere (FPA – frontal polar area, VLPFC – ventral lateral prefrontal cortex, VMC – ventral motor cortex, DLPFC – dorsolateral prefrontal cortex, DMC – dorsal motor cortex). fNIRS activity for the TBI-MF group is depicted with solid lines and controls with dashed lines. The oxy-Hb concentration in indicated in red and the deoxy-Hb in blue. All y-axis are presented as μ molar concentrations of oxy-Hb and deoxy-Hb. Significant effects are indicated for Group (G) or Group-Stroop interaction (GvS). ∗∗∗FDR adjusted p < 0.001, ∗∗FDR adjusted p < 0.01, ∗FDR adjusted p < 0.05. [file Image_1.TIF]

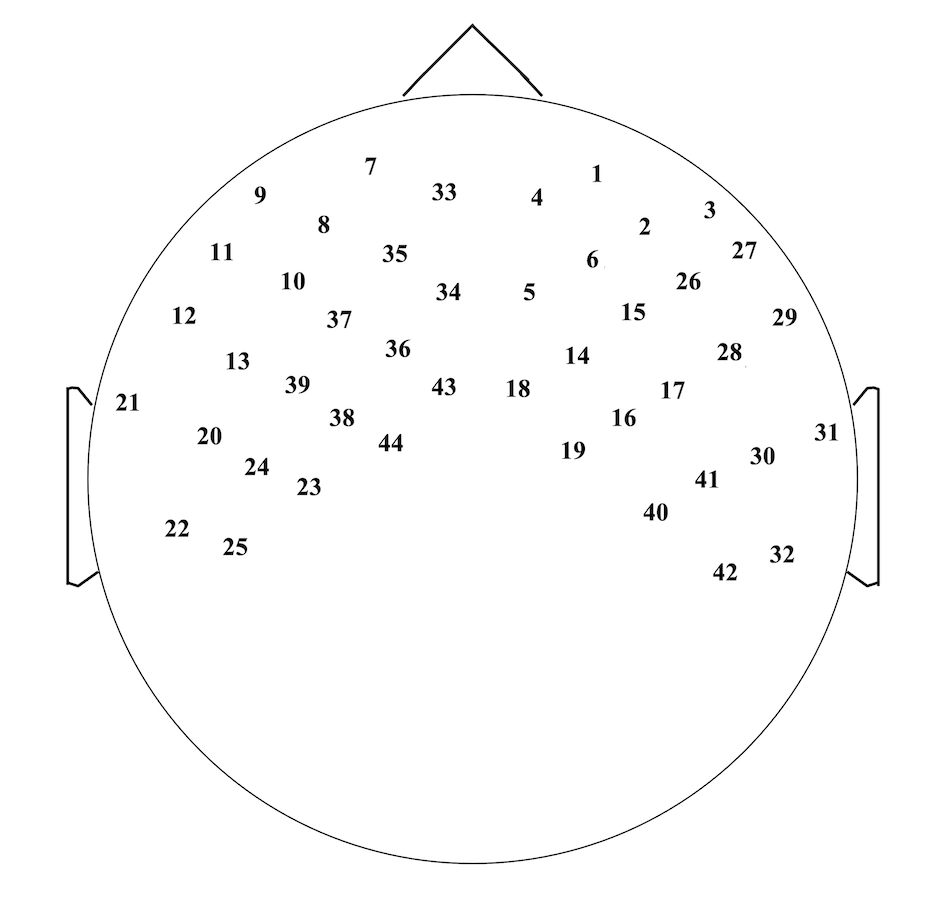

Supplement: FIGURE S2 — fNIRS channel locations viewed from above. [file Image_2.TIF]

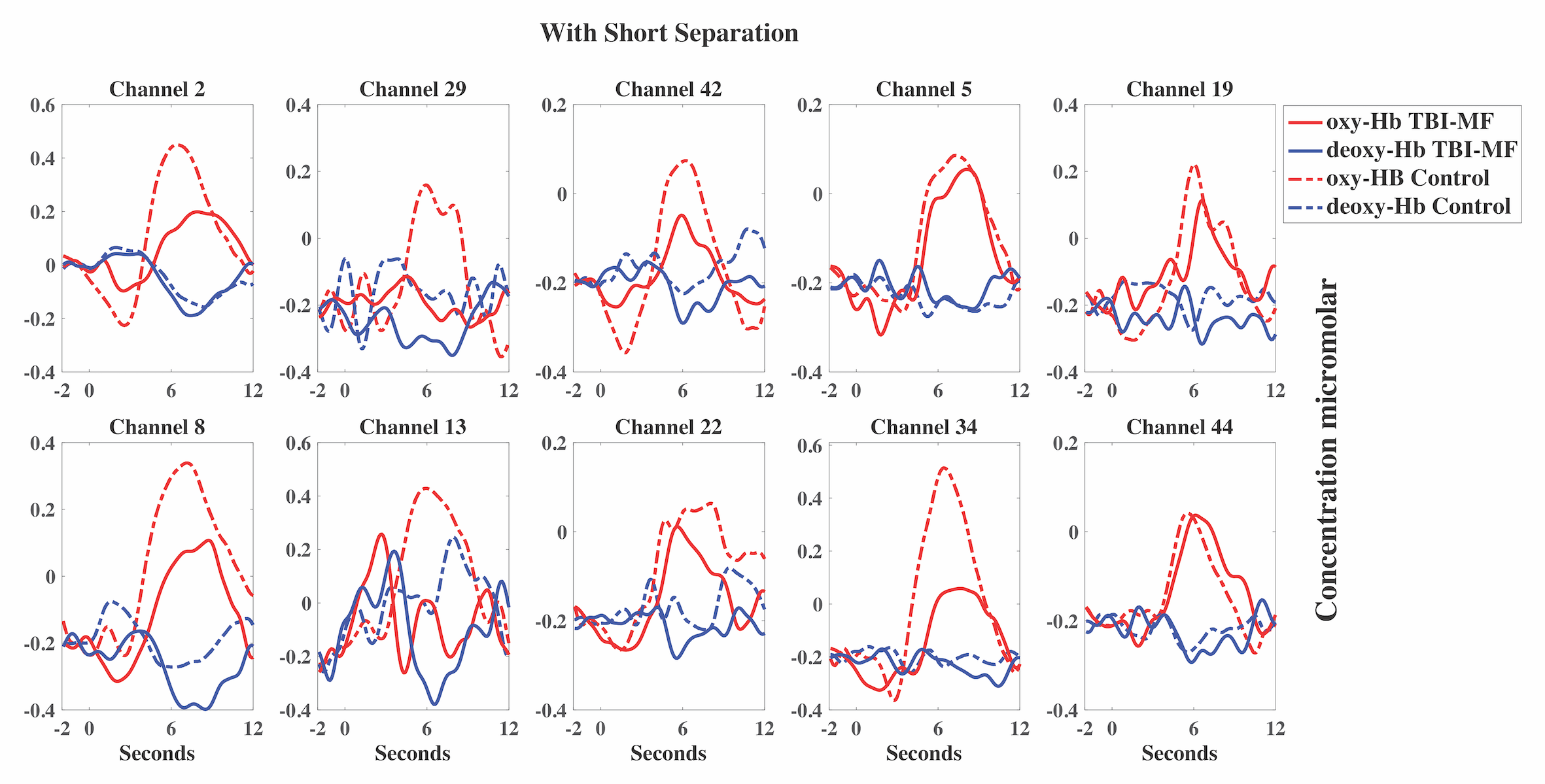

Supplement: FIGURE S3 — Oxy-Hb and deoxy-Hb concentration with short separation regression for Stroop incongruent trials in the Stroop-Simon test for one channel from each of the ten regions of interest. The TBI-MF group activity is depicted with the solid lines and the dashed lines represent the controls. Red lines indicate oxy-Hb concentrations and blue lines deoxy-Hb concentrations. [file Image_3.TIF]

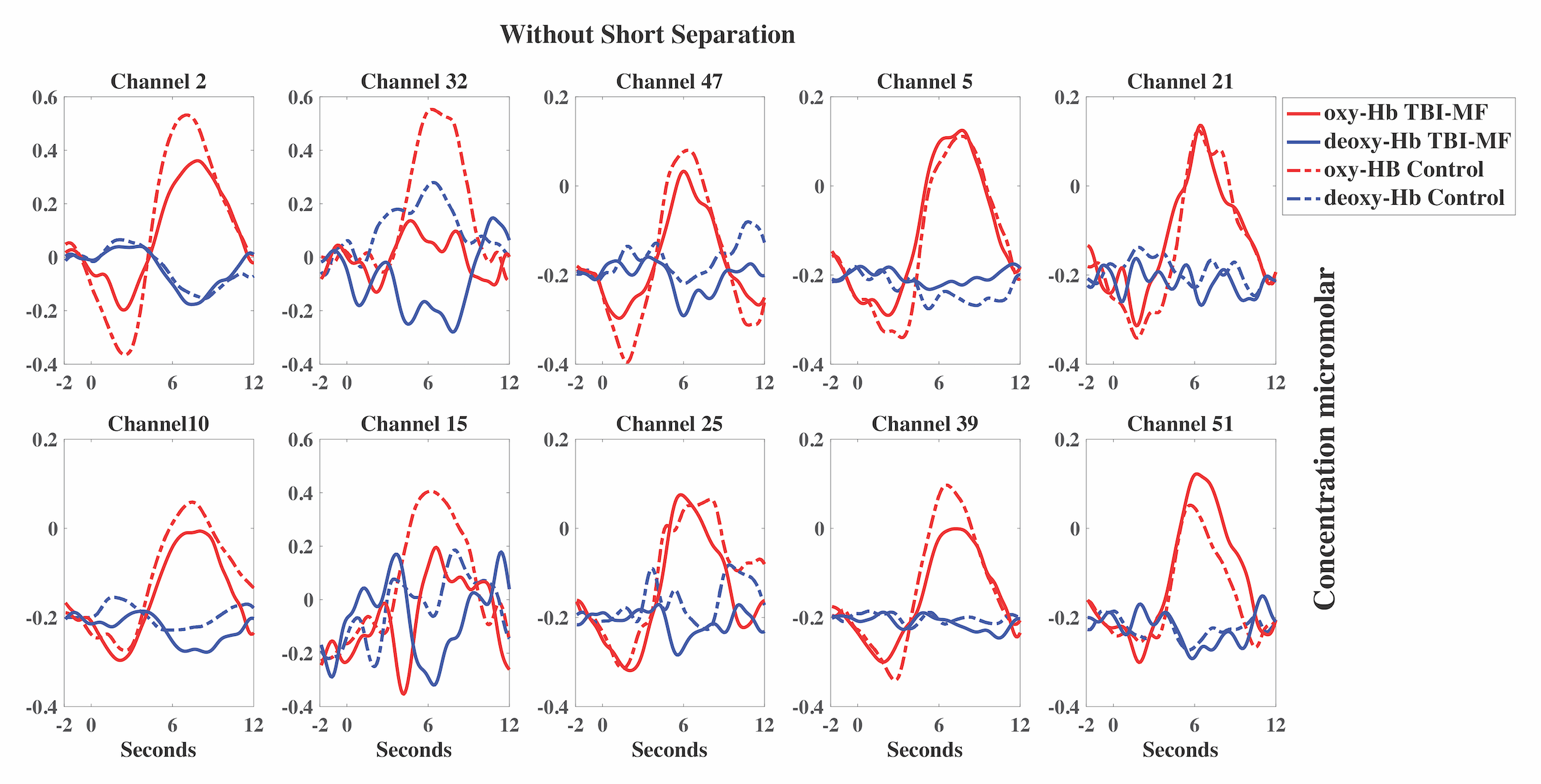

Supplement: FIGURE S4 — Oxy-Hb and deoxy-Hb concentration without short separation regression for Stroop incongruent trials in the Stroop-Simon test for one channel from each of the ten regions of interest. The TBI-MF group activity is depicted with the solid lines and the dashed lines represent the controls. Red lines indicate oxy-Hb concentrations and blue lines deoxy-Hb concentrations. [file Image_4.TIF]

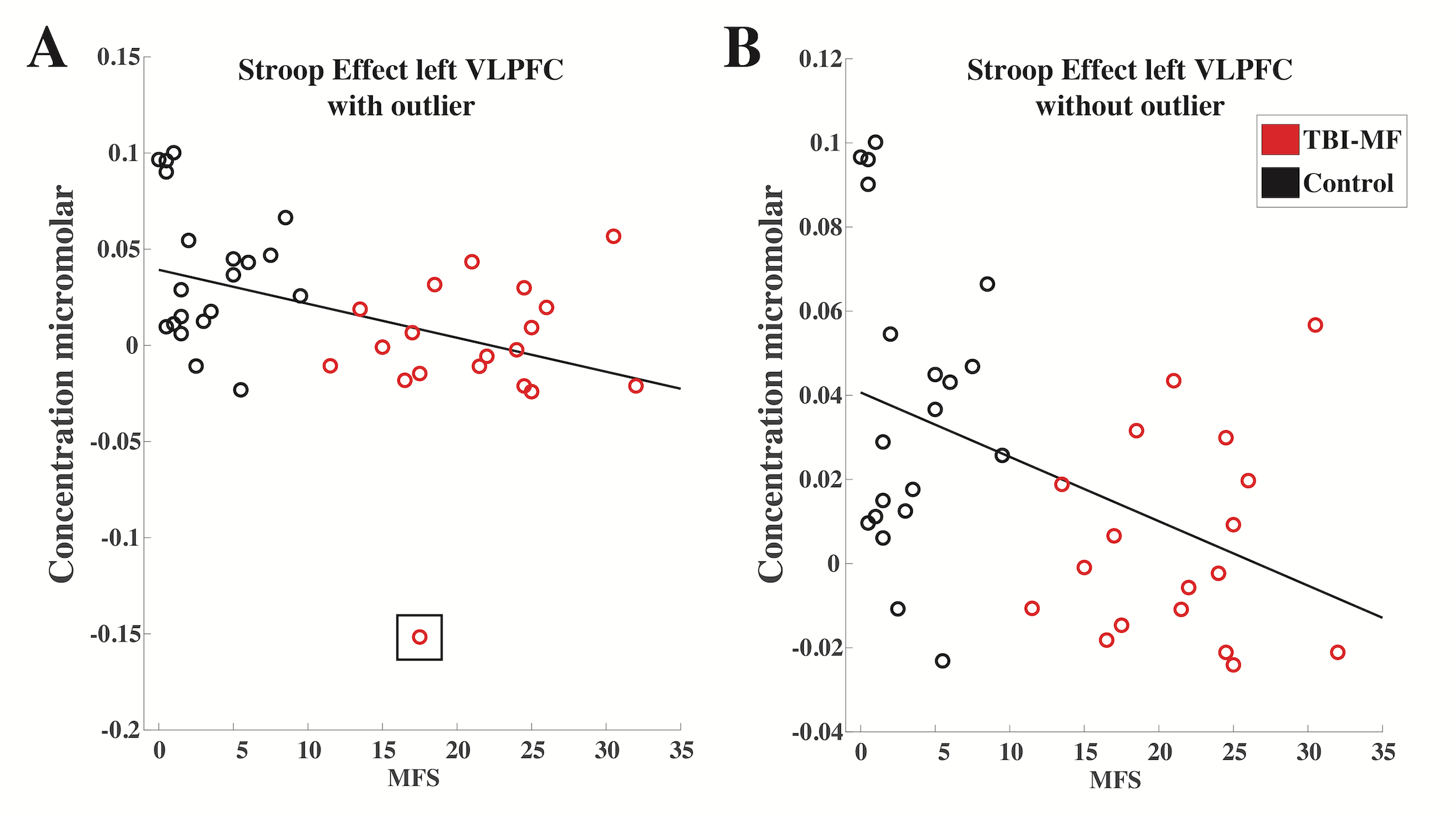

Supplement: FIGURE S5 — Pearson’s correlation between the mental fatigue scale (MFS) and Stroop effect in the left VLPFC. The Stroop effect is calculated in the fNIRS data as the oxy-Hb difference between incongruent and congruent trials in the Stroop dimension. (A) Correlation of r = −0.399, a possible outlier is highlighted with a rectangle. (B) Correlation of r = −0.442, the association is still present with the potential outlier removed. Both correlations are statistically significant with an FDR adjusted p < 0.05. [file Image_5.TIF]

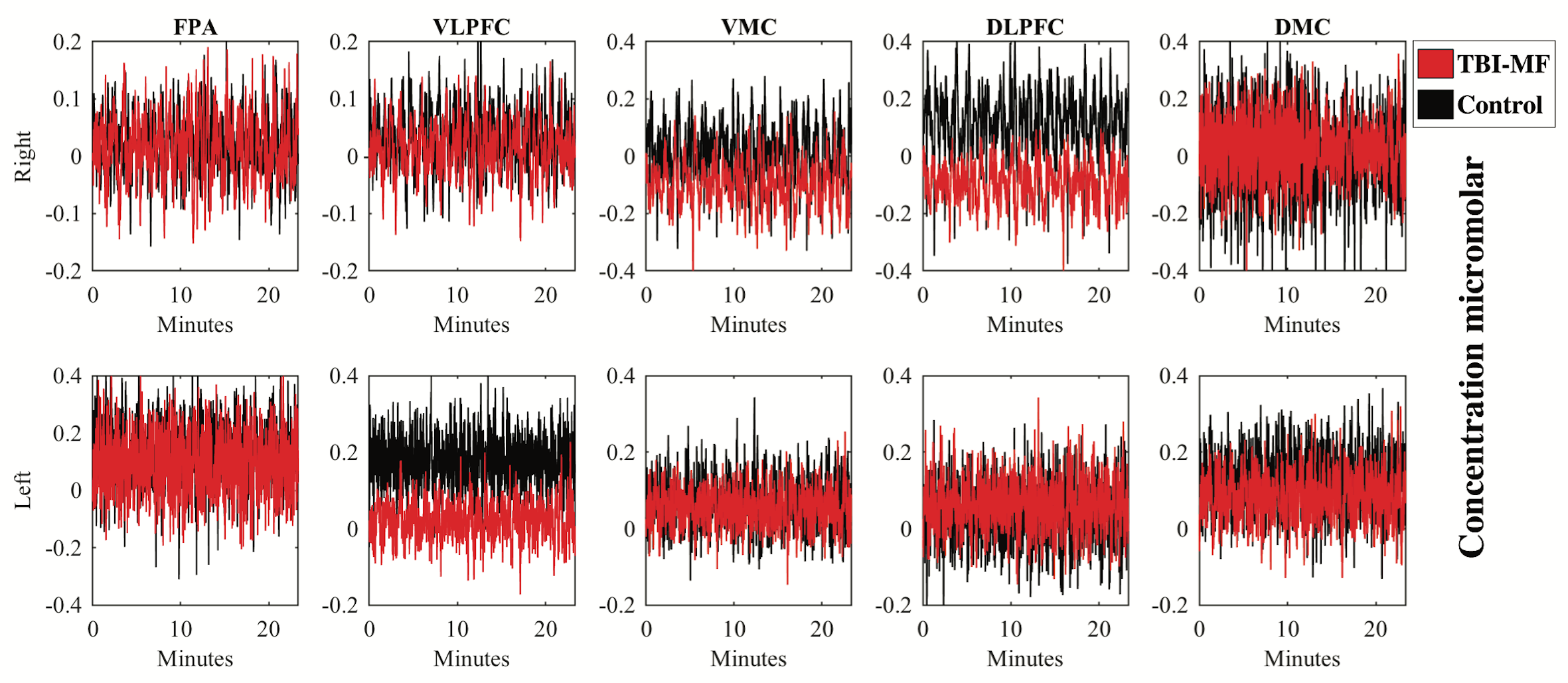

Supplement: FIGURE S6 — The oxy-Hb concentration for the complete first Stroop-Simon test session (23 min) for all ten ROI. The black curves indicate controls and the red curves the TBI-MF group. If the smaller oxy-Hb response of TBI-MF group were due to a maximum ceiling effect, we would predict a steady increase for the red curves. Since we did not observe such increase, we propose that the shortened stimulus-stimulus interval of 7-11 seconds had not affected the oxy-Hb levels of the TBI-MF group more than the controls. [file Image_6.TIF]
